# Degradation of Glyphosate in Soil Photocatalyzed by Fe_3_O_4_/SiO_2_/TiO_2_ under Solar Light

**DOI:** 10.3390/ijerph8041258

**Published:** 2011-04-21

**Authors:** Xuan Xu, Fangying Ji, Zihong Fan, Li He

**Affiliations:** 1 Key Laboratory of Three Gorges Reservoir Region’s Eco-Environment, Ministry of Education, Chongqing University, Chongqing 400045, China; E-Mails: jfy@cqu.edu.cn (F.J.); 20091702129@cqu.edu.cn (L.H.); 2 Institute of Urban Environment, Chinese Academy of Sciences, Xiamen 361021, China; E-Mail: zhfan@iue.ac.cn

**Keywords:** photocatalytic, solar light, titanium dioxide, glyphosate

## Abstract

In this study, Fe_3_O_4_/SiO_2_/TiO_2_ photocatalyst was prepared via a sol-gel method, and Fe_3_O_4_ particles were used as the core of the colloid. Diffraction peaks of Fe_3_O_4_ crystals are not found by XRD characterization, indicating that Fe_3_O_4_ particles are well encapsulated by SiO_2_. FTIR characterization shows that diffraction peaks of Ti-O-Si chemical bonds become obvious when the Fe_3_O_4_ loading is more than 0.5%. SEM characterization indicates that agglomeration occurs in the Fe_3_O_4_/SiO_2_/TiO_2_ photocatalyst, whereas photocatalysts modified by Fe_3_O_4_/SiO_2_ present excellent visible light absorption performance and photocatalytic activity, especially when the Fe_3_O_4_ loading is 0.5%. Photocatalytic degradation of glyphosate in soil by these photocatalysts under solar irradiation was investigated. Results show that 0.5% Fe_3_O_4_/SiO_2_/TiO_2_ has the best photocatalytic activity. The best moisture content of soil is 30%∼50%. Degradation efficiency of glyphosate reaches 89% in 2 h when the dosage of photocatalyst is 0.4 g/100 g (soil), and it increased slowly when more photocatalyst was used. Soil thickness is a very important factor for the photocatalytic rate. The thinner the soil is, the better the glyphosate degradation is. Degradation of glyphosate is not obviously affected by sunlight intensity when the intensity is below 6 mW/cm^2^ or above 10 mW/cm^2^, but it is accelerated significantly when the sunlight intensity increases from 6 mW/cm^2^ to 10 mW/cm^2^.

## 1. Introduction

Glyphosate is a kind of organophosphorus herbicide which is widely used in agriculture for weed control since it can inhibit the synthesis of aromatic amino acids in plants by inhibiting the synthase activity of 5-enol acetone shikimate-3-phosphate salt (EPSP) [1,2]. Due to its herbicidal effects, glyphosate has a strong adverse impact on all green plants if it enters the environment. Recently, the toxicity of glyphosate to other organisms has been recognized, and Dinehart and colleagues’ research shows that glyphosate is toxic to amphibians [3]. Langiano and colleagues found the same bad effects on fish [4]. Therefore, glyphosate removal or degradation has become a very important topic.

Research on glyphosate degradation has mainly focused on wastewater treatment processes [5–7], but in fact glyphosate in the environment is mainly found in the soil, so it is essential to develop effective methods that can remove glyphosate from soil. In recent years, photocatalytic technology, especially TiO_2_ photocatalysis, has been extensively developed in the field of organic pollutant treatment [8–11]. However, it is normally only applied in wastewater treatment, and is rarely utilized for *in situ* soil treatment [12,13]. Besides, the photocatalytic efficiency of TiO_2_ is limited by its wide band gap and radiation loss. The band gap of TiO_2_ (Anatase) is 3.2 eV. Only light below 385 nm can excite it. But this part of light is less than 4% of the total solar spectrum. Moreover, recombination of electrons and holes reduces the efficiency of radiation, and lead to the loss of photocatalytic activity. Thus, TiO_2_ photocatalysis using solar light is greatly limited. One effective way to solve these problems is to modify the photocatalyst with another semiconductor [14,15]. This kind of compound semiconductor photocatalyst has a wider range of excitation spectrum, and may even be excited by visible light [16,17]. In this study, a new kind of Fe_3_O_4_/SiO_2_/TiO_2_ photocatalyst was prepared with the purpose of using solar light and inhibiting the recombination of electrons and holes. *In situ* treatment of glyphosate in soil was performed using this Fe_3_O_4_/SiO_2_/TiO_2_ photocatalyst. The purpose of this research was to provide a new method and the basic data for the removal of organic pollutants in soil.

## 2. Materials and Methods

### 2.1. Chemicals

Tetra-*n*-butyl titanate, rhodamine B, tetraethyl orthosilicate, potassium bromate, potassium bromide acetic acid and sodium acetate were from Sinopharm Chemical Reagent Co., Ltd (China). Anhydrous ethanol, ferroferric oxide and ammonia were from Tianjin TianTai fine chemicals Co., Ltd (China). Tetra-*n*-butyl titanate was chemical grade. The other reagents were all of analytical grade.

### 2.2. Preparation of the Photocatalyst

Secondary pollution and the sunlight absorption ability of the photocatalyst must be considered for *in situ* treatment of soil. Fe_3_O_4_ is environmentally-friendly material, which can be excited by solar light because of its small band gap, so it was used to modify TiO_2_ to provide visible light response ability. SiO_2_ was used to encapsulate the Fe_3_O_4_ to prevent any decrease in catalytic activity when iron ion was incorporated into the TiO_2_ crystal structure.

#### 2.2.1. Preparation of Fe_3_O_4_/SiO_2_

Fe_3_O_4_ (0.005 g, 0.0125 g, 0.025 g, 0.05 g, 0.25 g and 0.5 g, respectively) was weighed into six beakers. Anhydrous ethanol (40 mL), tetraethyl orthosilicate (8 mL), ammonia (10 mL) and distilled water (1 mL) were added to each beaker. The resulting suspensions were stirred at room temperature for 5 h, and then dried at 105 °C. The resultant particles were Fe_3_O_4_/SiO_2_.

#### 2.2.2. Preparation of Fe_3_O_4_/SiO_2_/TiO_2_

The Fe_3_O_4_/SiO_2_ particles prepared with different amounts of Fe_3_O_4_ were suspended in anhydrous ethanol (40 mL) in one of six beakers and then acetic acid (15 mL), tetra-*n*-butyl titanate (21 mL) and distilled water (8.6 mL) were added to each of these six beakers. The mixtures were further stirred for 2 days, and then dried at 105 °C. The solid particles were ground to pass 500 mesh, and calcined at 500 °C (heating rate is 2 °C/min) in a muffle furnace. The resultant particles were Fe_3_O_4_/SiO_2_/TiO_2_, with theoretical Fe_3_O_4_ contents of 0.1%, 0.25%, 0.5%, 1%, 5% and 10%, respectively. TiO_2_ was prepared by the same method without any Fe_3_O_4_/SiO_2_.

#### 2.2.3. Photocatalyst Characterization

X-ray diffraction (XRD) patterns were collected in a XD-2 instrument (Persee, China) using Cu Kα radiation. Scanning electron microscopy (SEM) images were collected on an S-4800 field emission scanning electron microscope (Hitachi, Japan). Brunauer-Emmett-Teller (BET) surface areas were measured by nitrogen adsorption at 77.35 K on an ASAP-2010 adsorption apparatus (Micromeritics, USA).Fourier-transform infrared (FTIR) spectra were collected on an IRPrestige-21 type instrument (Shimadzu, Japan) running at 2 cm^−1^ resolution. The UV-vis spectra were collected on a UV-3010 UV-visible spectrometer (Hitachi, Japan) using BaSO_4_ as a reference. Glyphosate concentration was measured on a F-7500 fluorescence spectrometer (Hitachi, Japan).

#### 2.2.4. Degradation Experiment

The concentration of glyphosate standard stock solution was 1,000 mg/L. Soil for experiments was typical red loam, which was collected from the Banan District, Chongqing, China. The crushed soil was dried at 105 °C. Soil samples (50 g) and 4 mL/100 g (soil) of glyphosate solution were weighed into a glass dish (diameter 120 mm). Moisture content of this mixture was adjusted to a predetermined value using distilled water. Then a certain amount of photocatalyst was added and mixed well. The dish was placed under sunlight for 2 h. To keep the soil moisture constant, a certain amount of distilled water was added every 15 min by weighing the dish to calculate the water volume, while mixing the sample at the same time. After 2 h irradiation all the soil was suspended in distilled water, and separated by centrifugation after 10 min washing. This process was repeated four times, and then the solution volume was adjusted to 500 mL sing distilled water. The concentration of glyphosate was determined by a fluorescence quenching method [18]. Three parallel experiments were carried out for each experiment, and the results were the average of these three experiments. The glyphosate degradation by photolysis used for comparison was carried out without any photocatalyst.

## 3. Results and Discussion

### 3.1. Photocatalysts Characterization

#### 3.1.1. XRD Characterization

**Figure 1.**
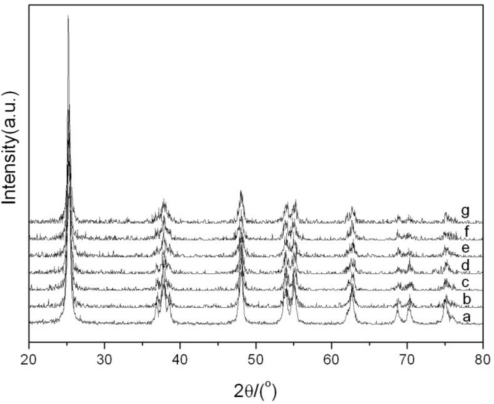
XRD spectra of photocatalysts: (**a**) TiO_2_; (**b**) 0.1%Fe_3_O_4_/SiO_2_/TiO_2_; (**c**) 0.25%Fe_3_O_4_/SiO_2_/TiO_2_; (**d**) 0.5%Fe_3_O_4_/SiO_2_/TiO_2_; (**e**) 1%Fe_3_O_4_/SiO_2_/TiO_2_; (**f**) 5%Fe_3_O_4_/SiO_2_/TiO_2_; (**g**) 10%Fe_3_O_4_/SiO_2_/TiO_2_.

The XRD patterns of photocatalysts are compared in Figure 1. Only the diffraction peaks of typical anatase TiO_2_ were observed with 2*θ* at 25.3°, 37.9°, 48.2°, 54.0°, 55.1°, 62.6°, 68.8°, 70.3°and 75.2°. Diffraction peaks of titanate and Fe_3_O_4_ crystal didn’t appear. This indicates that the Fe_3_O_4_ is well encapsulated by SiO_2_, and no interaction between Fe_3_O_4_ and TiO_2_ occurs.

#### 3.1.2. SEM Characterization

**Figure 2.**
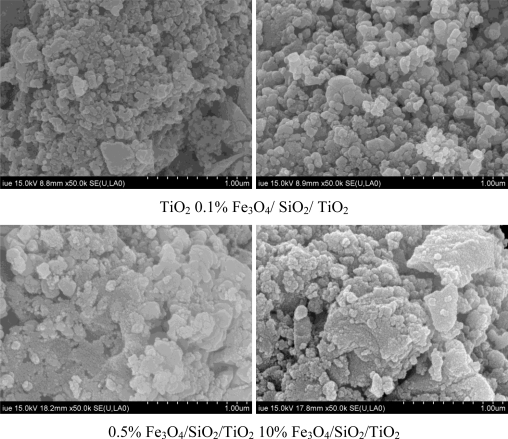
SEM photographs of photocatalysts with different Fe_3_O_4_ loadings.

Figure 2 shows the scanning electron microscopy (SEM) images of TiO_2_ and Fe_3_O_4_/SiO_2_/TiO_2_ with 0.1%, 0.5%, 10% Fe_3_O_4_, respectively. As shown in the figure, the crystal sizes of these four catalysts were all about 1 μm, but agglomeration of catalysts ocurred when Fe_3_O_4_/SiO_2_ was added, and this phenomenon became more apparent as the Fe_3_O_4_/SiO_2_ content increased. The Fe_3_O_4_/SiO_2_ particles play a vital role and are encapsulated by Ti(OH)_4_ constantly during hydrolysis, so the particle size of the Fe_3_O_4_/SiO_2_/TiO_2_ catalyst ultimately increases and its dispersion properties weaken. SEM characterization shows that excess Fe_3_O_4_/SiO_2_ in the Fe_3_O_4_/SiO_2_/TiO_2_ photocatalyst may lead to a sharp decrease of photocatalyst dispersion properties.

The BET surface area, average pore diameter and total pore volume of all catalysts are listed in Table 1. Characterization results showed that Fe_3_O_4_ lowered BET surface area and total pore volume while the average pore diameter of Fe_3_O_4_/SiO_2_/TiO_2_ photocatalyst increased. These changes are also due to dispersion property variation caused by the addition of Fe_3_O_4_.

#### 3.1.3. FTIR Characterization

**Figure 3.**
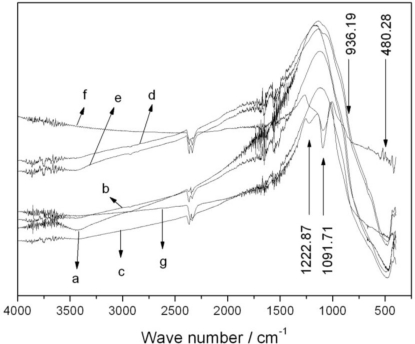
FTIR spectra of photocatalysts: (**a**) TiO_2_; (**b**) 0.1%Fe_3_O_4_/SiO_2_/TiO_2_; (**c**) 0.25%Fe_3_O_4_/SiO_2_/TiO_2_; (**d**) 0.5%Fe_3_O_4_/SiO_2_/TiO_2_; (**e**) 1%Fe_3_O_4_/SiO_2_/TiO_2_; (**f**) 5%Fe_3_O_4_/SiO_2_/TiO_2_; (**g**) 10%Fe_3_O_4_/SiO_2_/TiO_2_.

Functional groups on different photocatalysts were confirmed by Fourier-transform infrared spectroscopy. The FTIR characterization results are presented in Figure 3. As shown in the spectra the Si-O-Si asymmetric stretching vibration peaks [19] around 1,091.71 cm^−1^ and 1,222.67 cm^−1^ appeared when the Fe_3_O_4_ load reached 0.5%. This demonstrated that a certain amount of SiO_2_ had formed on the catalysts, and these peaks became increasingly apparent as the Fe_3_O_4_ load continued to increase. The asymmetric stretching vibration [20] of Ti-O-Si around 936.19 cm^−1^ appeared when the Fe_3_O_4_ loading reached 1%. This peak indicated that certain amount of Si had replaced Ti in the TiO_2_ lattice. The peak at 480.28 cm^−1^ was assigned to Ti-O stretching vibrations. There was no evidence for any interaction between Fe and Ti in the spectra. It shows that Fe_3_O_4_ is encapsulated by SiO_2_ very well, therefore the photocatalytic activity of Fe_3_O_4_/SiO_2_/TiO_2_ is not reduced by this interaction. Because of the role of capturing electrons, less Ti-O-Si will promote isolation of hole-electron pairs, enhancing the photocatalytic activity. However, excessive Ti-O-Si will produce recombination centers for holes and electrons, and reduce the photocatalytic activity.

#### 3.1.4. UV-Vis Characterization

**Figure 4.**
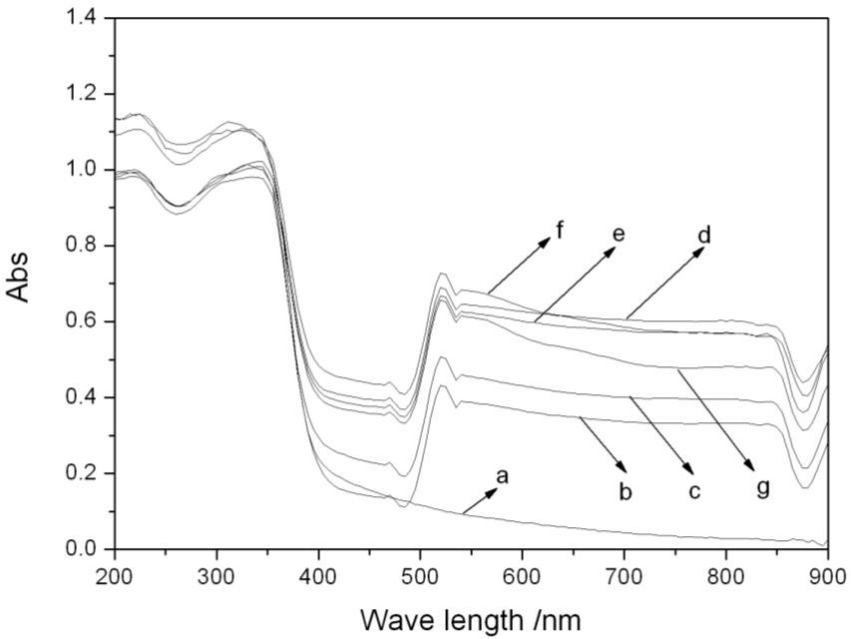
UV-Vis DRS of photocatalysts: (**a**) TiO_2_; (**b**) 0.1%Fe_3_O_4_/SiO_2_/TiO_2_; (**c**) 0.25%Fe_3_O_4_/SiO_2_/TiO_2_; (**d**) 0.5%Fe_3_O_4_/SiO_2_/TiO_2_; (**e**) 1%Fe_3_O_4_/SiO_2_/TiO_2_; (**f**) 5%Fe_3_O_4_/SiO_2_/TiO_2_; (**g**) 10%Fe_3_O_4_/SiO_2_/TiO_2_.

The UV-Vis absorption properties of different catalysts are shown in Figure 4. In this figure a clear absorption peak can be found in the visible region (λ ≥ 420 nm) on every catalyst spectrum with Fe_3_O_4_. All spectra still maintained the absorption characteristics of TiO_2_ in the ultraviolet region, indicating that there is no interaction between TiO_2_ and Fe_3_O_4_, because of the separation effect of SiO_2_. UV-Vis characterization demonstrates that photocatalysts with Fe_3_O_4_ have excellent photoluminescence capability in both the visible and ultraviolet regions. These photocatalysts may exhibit higher photocatalytic activity than TiO_2_ without Fe_3_O_4_/SiO_2_. It can also be seen from this figure that the Fe_3_O_4_ loading affected the visible light absorption properties of the catalysts significantly. Absorption capacity was enhanced by increasing Fe_3_O_4_ loading when the Fe_3_O_4_ loading was less than 0.5%, then it weakened when the Fe_3_O_4_ loading was more than 0.5%. The 0.5% Fe_3_O_4_/SiO_2_/TiO_2_ has the strongest absorption capacity and thus may have the best photocatalytic activity.

### 3.2. Factors Affecting Glyphosate Degradation

#### 3.2.1. Fe_3_O_4_ Loading

**Figure 5.**
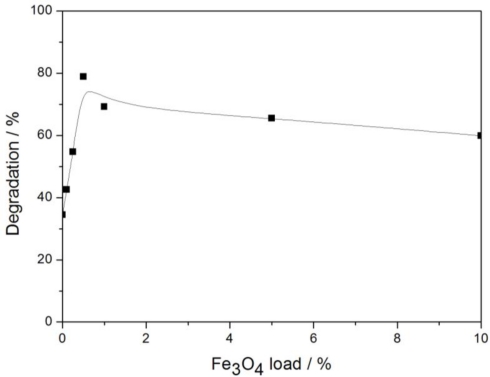
Effect of Fe_3_O_4_ load on glyphosate degradation.

Six samples were prepared with 50 g soil, 0.2 g photocatalysts and 2 mL glyphosate standard stock solution. The photocatalyst in each sample had different Fe_3_O_4_ loading. The moisture content of samples was adjusted to 40%. Experimental results after 2 h irradiation (sunlight intensity was 11.88 mW/cm^2^) are shown in Figure 5. It can be seen that catalysts with Fe_3_O_4_ had better activity than TiO_2_. Fe_3_O_4_ greatly enhances the efficiency of visible light absorption, so Fe_3_O_4_/SiO_2_/TiO_2_ catalysts show excellent photocatalytic activity under sunlight irradiation. Using Fe_3_O_4_ as photocatalyst, the degradation of glyphosate was only 53.04%, which was much lower than with 0.5% Fe_3_O_4_/SiO_2_/TiO_2_ where the degradation was 78.84%. It indicates that Ti-O-Si bond in Fe_3_O_4_/SiO_2_/TiO_2_ catalyst can reduce the probability of the hole-electron pair recombination, thus improving the photocatalytic activity of the catalyst.

Degradation of glyphosate increased from 34.42% to 78.84% when the Fe_3_O_4_ load was varied from 0 to 0.5%, correspondingly. Degradation efficiency declined when more than 0.5% Fe_3_O_4_ was loaded. The Fe_3_O_4_ in the catalysts has two kinds of effects. On one hand, it can enhance the visible sunlight absorption properties of the catalyst. On the other hand, the interaction between Si and Ti has a negative effect on the photocatalytic activity when the Fe_3_O_4_ loading is excessive. Which aspect is more important depends on the amount of Fe_3_O_4_. Characterization through FTIR shows that Ti-O-Si bonds become apparent in the catalyst with 1% Fe_3_O_4_ loading, which illustrates that Ti-O-Si has become a recombination center for holes and electrons, so photocatalyts with 1%, 5% and 10% Fe_3_O_4_ decrease the photocatalytic activity. 0.5% Fe_3_O_4_/SiO_2_/TiO_2_ was thus chosen for the subsequent experiments.

#### 3.2.2. Moisture Content

**Figure 6.**
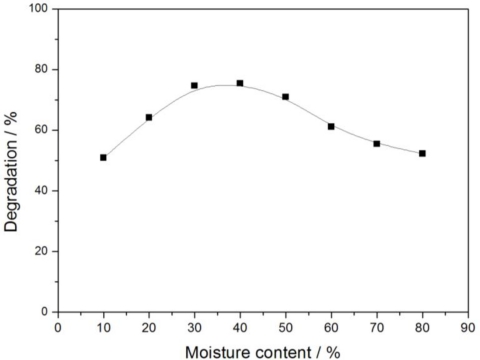
Effect of moisture content on glyphosate degradation.

Eight samples were prepared with 50 g soil, 0.2 g 0.5% Fe_3_O_4_/SiO_2_/TiO_2_ photocatalyst and 2 mL glyphosate standard stock solution. The moisture content was adjusted to a reference value. Experimental results are shown in Figure 6 (sunlight intensity was 11.73 mW/cm^2^). In view of Figure 6, degradation of glyphosate was not ideal when the moisture content was lower than 30% or higher than 50%. Degradation was basically at a higher level with a moisture content ranging from 30% to 50%. The moisture content of samples affects the glyphosate distribution among the water, soil and air phases in soil. In addition, the moisture content also affects the amount of free radical formation on the catalyst surface. Glyphosate cannot diffuse to the catalyst surface fast when the moisture content is lower than 30%. Only if the glyphosate is adsorbed on the surface of photocatalysts, can it be degraded because the lifetime of free radicals is reportedly very short, and these free radicals are practically produced on surface of photocatalysts. Therefore, a low moisture content hinders the degradation of glyphosate. On the contrary, O_2_ cannot diffuse to the surface of photocatalysts, when the moisture content is higher than 50%. This effect results in a significant decrease in the production of free radicals, because O_2_ is one of the most important substances in the free radical generation process. Therefore too much water in soil also leads to a lower removal rate. The most efficient degradation of glyphosate ocurrs when the moisture content is between 30% and 50% for the diffusion of pollutants and O_2_ balance. The moisture content in subsequent experiments was set at 40%.

#### 3.2.3. Photocatalyst Dosage

**Figure 7.**
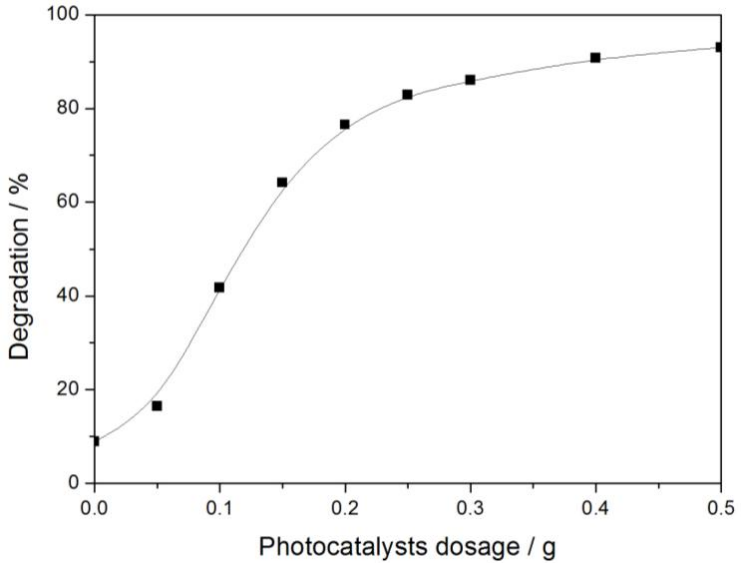
Effect of photocatalyst dosage on glyphosate degradation.

A certain amount of 0.5% Fe_3_O_4_/SiO_2_/TiO_2_ catalyst and 2 mL glyphosate standard stock solution were separately added to nine samples. The moisture content of all samples was adjusted to 40%. Degradation of glyphosate irradiated 2 h by sunlight is shown in Figure 7 (sunlight intensity was 11.79 mW/cm^2^). As seen in the figure, it can be concluded that an increase in photocatalyst dosage accelerated the rate of degradation of glyphosate. Degradation was proportional to the photocatalyst dosage when the latter was less than 0.2 g. It increased rapidly. Nevertheless, further photocatalysts led to a slow increase of degradation. There is a maximum value of glyphosate diffusions rate because its concentration in the sample is limited. When this maximum value is reached, excess photocatalyst is useless. Glyphosate concentration is the limiting factor in the reaction under these conditions, so the reaction rate tends to remain constant.

#### 3.2.4. Soil Thickness

**Figure 8.**
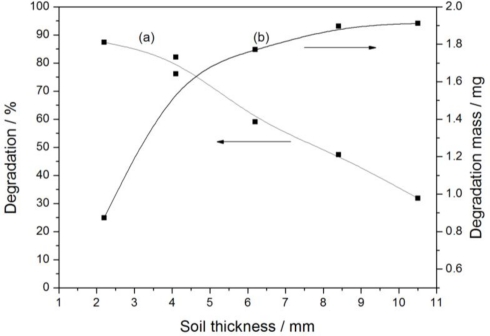
Effact of soil thickness on glyphosate degradation: (**a**) Degradation curve; (**b**) Degradation mass curve.

Soil samples (25 g, 50 g, 75 g, 100 g and 150 g) were placed separately in five glass dishes (120 mm diameter). Photocatalyst (0.5% Fe_3_O_4_/SiO_2_/TiO_2_) dosage and glyphosate standard stock solution amounts were 0.4 g/100 g (soil) and 4 mL/100 g (soil), respectively. Moisture content of soil was raised to 40%. The thicknesses of these samples were 2.2 mm, 4.1 mm, 6.2 mm, 8.4 mm and 10.5 mm after tiling. Degradation of glyphosate is shown in Figure 8(a) (sunlight intensity was 11.97 mW/cm^2^). In view of this figure, we can simply conclude that the thicker the sample was, the lower the degradation rate was, as 87.33% glyphosate was removed after 2 h irradiation when the soil thickness was 2.2 mm., whereas on the contrary, the removal rate was reduced to 38.21% when thickness was 10.5 mm. This is because the sunlight cannot reach deep inside the soil, so the necessary conditions for the photocatalytic degradation in this part of soil are absent and suggesting that the photocatalytic reaction only occurs in the surface part of soil and degradation decreases as the soil layer becomes thicker.

However, Figure 8 (b) shows that the total mass degradation of glyphosate in 2 h kept increasing despite the fact the degradation went down. Photocatalysis occurs in all levels of the soil when the soil thickness declines, because sunlight can penetrate all the layers. Under these conditions, increasing the thickness can promote the amount of glyphosate involved in photocatalysis, so the mass degradation increases rapidly. When the soil thickness is beyond sunlight exposure capacity, no more soil and glyphosate are involved in photocatalytic reaction, so glyphosate degradation mass in 2 h tends to a fixed quantity. It is shown in Figure 8 (b) that the maximum degradation amount within 2 h in this system is about 1.9 mg.

#### 3.2.5. Light Intensity

**Figure 9.**
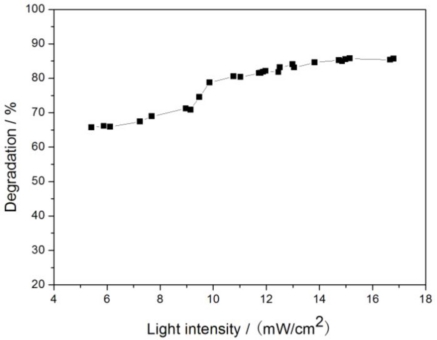
Effect of irradiation intensity on glyphosate degradation.

All samples were prepared with 50 g soil, 0.2 g 0.5% Fe_3_O_4_/SiO_2_/TiO_2_ photocatalyst and 2 mL glyphosate standard stock solution. Moisture content of all samples was adjusted to 40%. These samples were irradiated under different weather conditions. According to the light intensity data, the experimental results shown in Figure 9 suggest that the glyphosate degradation rate increased slowly with sunlight intensity. The removal rate was 67% when the light intensity was about 6 mW/cm^2^, and it reached 86% when the light intensity was about 16 mW/cm^2^. Glyphosate was degraded faster when the light intensity exceeded 10 mW/cm^2^. We tentatively interpret this phenomenon as a consequence of the temperature change of the soil. Generally, when it is cloudy and the temperature is lower when the irradiation intensity is below 10 mW/cm^2^. Higher sample temperatures are conductive to the photocatalytic degradation.

## 4. Conclusions

TiO_2_ was modified by SiO_2_ encapsulated Fe_3_O_4_. The resulting Fe_3_O_4_/SiO_2_/TiO_2_ photocatalyst exhibited excellent visible light photocatalytic ability. The Fe_3_O_4_ loading affected the structure and photocatalytic activity of photocatalysts, and the best loading amount was determined to be 0.5%. Using Fe_3_O_4_/SiO_2_/TiO_2_ photocatalyst to degrade glyphosate in soil, the photocatalytic reaction was affected by the moisture content of soil, photocatalyst dosage, soil thickness and solar light intensity. The degradation rate reached the fastest value when the moisture content was 40%, photocatalyst dosage was 0.4 g/100 g (soil), soil thickness was less than 4.1 mm, and sunlight intensity exceeded 10 mW/cm^2^.

## Figures and Tables

**Table 1.** BET surface area, average pore diameter and total pore volume of photocatalysts.

**Sample**	**BET specific surface area (m^2^/g)**	**Average pore diameter (nm)**	**Total pore volume (cm^3^/g)**
TiO_2_	127.38	19.56	0.2731
0.1% Fe_3_O_4_/ SiO_2_/ TiO_2_	84.39	24.47	0.2418
0.25% Fe_3_O_4_/ SiO_2_/ TiO_2_	76.91	28.72	0.2273
0.5% Fe_3_O_4_/ SiO_2_/ TiO_2_	64.27	31.67	0.2080
1% Fe_3_O_4_/ SiO_2_/ TiO_2_	50.49	37.83	0.1843
5% Fe_3_O_4_/ SiO_2_/ TiO_2_	41.08	40.51	0.1694
10% Fe_3_O_4_/ SiO_2_/ TiO_2_	32.82	49.17	0.1467
